# Supplementary material for: Anti-SARS-CoV-2 IgG and IgA antibodies in COVID-19 convalescent plasma do not enhance viral infection
Source: PLoS One. 2022 Mar 8;17(3):e0257930. doi: 10.1371/journal.pone.0257930 (PMC8903276; doi:10.1371/journal.pone.0257930)
Supplement: S1 File — (DOCX) [file pone.0257930.s001.docx]

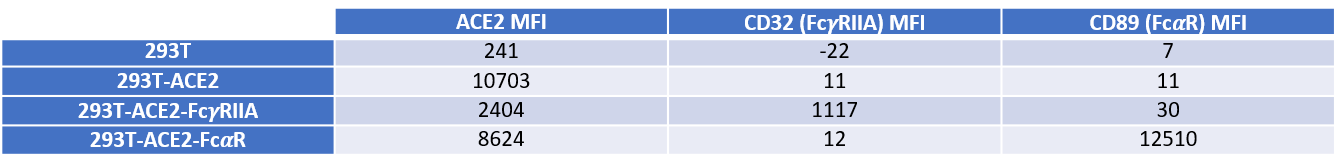

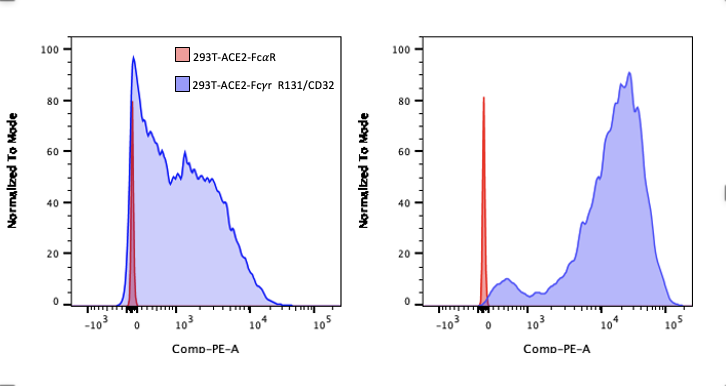

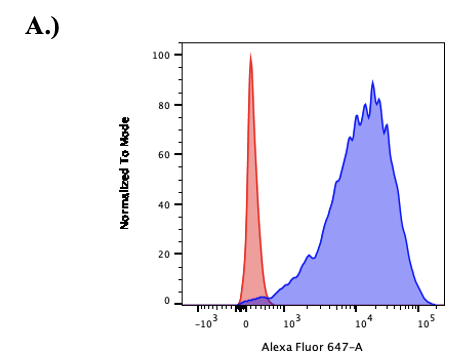

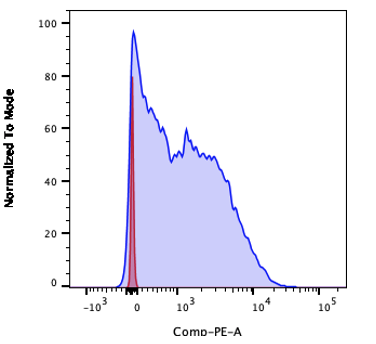


293T-ACE2

293T-ACE2-Fc𝛼R/CD89

293T

293T-ACE2

293T-ACE2

293T-ACE2-Fc𝛾R R131/CD32

**Flow cytometry analysis of 293T cell expression levels of ACE2 and CD32.** 293T cells were transduced with retroviral

particles carrying the pQCXIP vector encoding the gene for the human ACE2 protein. After confirmation of ACE2 expression,

293T-ACE2 cells were transduced with MLV particles carrying the pQCXIH or pQCXIP vector encoding the gene for

Fc𝛼R/CD89 or Fc𝛾RIIA-R131/CD32, respectively. After antibiotic selection, 293T and 293T-ACE2 cells were stained with

monoclonal antibodies against CD89 or CD32 followed by a rabbit polyclonal anti-human ACE2 antibody and a AF488 or

AF647-conjugated secondary anti-rabbit IgG antibody. **(A)**. The table shown contains the Mean Fluorescence Intensity (MFI)

values measured by flow cytometry for ACE2, CD89, and CD32 staining on all cell lines used in this experiment **(B)**.

**B.)**

**A.)**

ACE2

CD32

CD89
